# Understanding carers’ fall concern and their management of fall risk among older people at home

**DOI:** 10.1186/s12877-019-1162-7

**Published:** 2019-05-24

**Authors:** Seng Giap Marcus Ang, Anthony Paul O’Brien, Amanda Wilson

**Affiliations:** School of Nursing and Midwifery, Faculty of Health and Medicine, University of Newcastle, Callaghan, NSW 2308 Australia

**Keywords:** Carer, Older people, Fall concern, Fall risk, Fear of falling, Qualitative research

## Abstract

**Background:**

Many older people (care recipients) experience long-term psychological distress due to the fear of falling again. Falls can affect carers due to concerns about their care recipients falling. Understanding carers’ fall concern is crucial to determine if carers are coping with the provision of care or have adequate knowledge and support in preventing their care recipients from falling at home.

**Methods:**

A descriptive qualitative study was conducted to explore carers’ concern about their care recipients being at risk of falling and their management of fall risk at home. Twenty-two carers were recruited from two research registers and a large tertiary hospital in a regional centre of Australia. Carers were interviewed face-to-face, or by telephone using a semi-structured interview guide about their fall concern. The data was analysed using an inductive content analysis method.

**Results:**

Eight major themes emerged from the interviews. Four themes described key factors influencing carers’ fall concern which include: 1) carers’ perception of fall and fall risk, 2) care recipients’ behaviour and attitude towards fall risk, 3) care recipients’ health and function, and 4) care recipients’ living environment. Another four themes described the management of care recipients’ fall risk which include: 5) fall prevention strategies used, 6) risk of preventing falls, 7) support from family and friends, and 8) support from healthcare professionals.

**Conclusions:**

The findings from this qualitative study provide an insight into the carers’ awareness of fall risk, knowledge, and the availability of support in preventing their care recipients from falling at home. Healthcare professionals are encouraged to include carers and address their fall concern to improve fall prevention programmes for care recipients at risk of falling at home.

## Background

Falls are a serious problem among older people which result in injuries, deaths, and long-term psychological consequences [1]. Globally, 28 to 35% of people aged 65 and above fall each year with the rate of falls increasing with age and frailty [2]. There were 98,704 older people hospitalised for injuries sustained by falling in Australia between 2012 and 2013; an increase of 24,000 cases over 10 years [3]. A common complication of a fall is the fear of falling again, which affects between 3 and 85% of the older people [4]. This psychological concern about falling can pose a significant threat to an older person’s autonomy resulting in self-imposed activity restriction and a loss of confidence in their ability to ambulate safely [5].

Like older people (care recipients), falls can also affect their carers [6]. Previous research has highlighted that after a fall has occurred, most carers experience increased concern about their care recipients falling again [7, 8]. This concern is significantly associated with increased psychological distress, social restriction, and caregiving burden among carers [8–11]. Concerns about their care recipients falling have resulted in many carers adopting various strategies to prevent falls, including, increased vigilance and not leaving them alone at home [12–15]. However, such approaches often lead to carers not having enough time for themselves, or to fulfil other social and work duties.

Carers are an essential part of the caring support network for older people which enables them stay at home longer, delaying institutional care if they (older people) should deteriorate with their physical health [11]. Previous fall prevention programmes that included carers have found significant improvement in fall risk awareness and knowledge among cancer patients at risk of falling [16], and better balance and fall efficacy among older people living with dementia [17]. However, carers who are overly supportive and prevent their care recipients from engaging in their daily activities, can inadvertently increase care recipients’ dependence [18]. Such outcomes have been attributed to the loss of confidence in their care recipients’ balance, and the need to constantly provide assistance to the care recipients in their daily activities also restrict carers from participating in their personal or social activities [18].

Carers are closely involved in the care of older people and are important for the successful implementation of fall prevention strategies at home. However, there is no published research on the perceived impact of fall risk among older people (with and without falls) on carers. Previous qualitative studies of carers’ perspectives have focused on care recipients who have fallen or diagnosed with medical conditions such as Parkinson’s disease or cognitive impairment which places them at a higher risk of falling [12, 14, 15]. In addition, there is no evidence regarding the common fall prevention strategies used by carers looking after care recipients from the general population of older people.

The aims of this study were to explore 1) carers’ concern for the risk of falling among care recipients, known as carers’ fall concern, and 2) their management of fall risk at home. The outcomes of this study have implications for future improvements of fall prevention programmes targeting carers’ fall concern, while reducing falls and fall risk of the care recipients living at home under their care. The findings from this study will facilitate healthcare professionals to assess how carers are coping with the provision of care and if they have appropriate access to support, or relevant knowledge in preventing their care recipients from falling at home.

## Methods

### Design

This study was conducted between October 2017 and February 2018. A descriptive qualitative design was applied because little is known about the topic of carers’ fall concern [19]. The method of data collection and analysis in a descriptive qualitative study allows researchers to stay close to the data by presenting a comprehensive summary of everyday events using simple language to increase agreement among researchers and carers [20].

### Participants

Participants were recruited from three study sites: 1) Hunter Medical Research Institute (HMRI) (research register and Facebook Page), 2) Carers New South Wales (Carers NSW) (membership register), and 3) the Rheumatology outpatient clinic at a large regional teaching hospital in NSW. The study sites provided access to carers living in metropolitan and regional New South Wales, Australia [21]. At HMRI and Carers NSW, study invitations were sent to the registry members by the research coordinators. Participants who were keen to participate responded to the HMRI coordinator by completing a study response form containing their contact information, which was then forwarded to the researchers for contacting purposes. Other interested participants who received study information from the HMRI Facebook page or Carers NSW contacted the researchers directly regarding their interest. In the outpatient clinic, the rheumatology nurses assisted with distributing the study flyers to carers who had accompanied their family members for follow-up appointments. Interested participants could either contact the researchers directly or approach the nurses who would then refer them to one of the researchers.

The inclusion criteria consisted of people currently providing care for a family member or friend aged 60 years and over and living at home. The carer did not have to live with the care recipient, however participants who were aged below 18 years old, or working as professional or paid carers were excluded. As the study intended to explore the fall concern of the general population of carers, the recruitment was not restricted to carers looking after care recipients with an increased risk of falling. In addition, carers of care recipients with and without the use of professional support services were also included. The study was approved by the Hunter New England Health Human Research Ethics Committee [17/09/20/4.03]. All participants were provided with the study details and written consent was taken before the interview.

Twenty-two participants were recruited using purposive sampling, which involved a deliberate selection of participants to obtain a complete understanding of carers’ fall concern [19]. The carers differed in terms of age, gender, and the care recipients’ frequency of falls and severity of injury sustained. These factors have been associated with older people’s fear of falling which could potentially affect carers’ fall concern [5]. The relationship between carer and care recipient was also considered as the type of caregiving relationship, such as carers caring for their spouses versus carers caring for their parents may affect the fall prevention strategies used [22]. The recruitment continued until no new themes were emerging from the qualitative data.

### Data collection

Face-to-face or telephone interviews were conducted using a semi-structured interview guide by one researcher (MA, first author) who was a registered nurse with experience in caring for older people. The face-to-face interviews occurred at a convenient and quiet place, such as an unused office, or quiet area close to the outpatient department. The option of telephone interview was provided to encourage participation from carers who were unable to leave their care recipients alone at home because they were afraid of their care recipients falling when unsupervised. Examples of the interview questions were: 1) can you tell me about your concerns caring for your family member and their risk of falling, 2) what helps you care for a family member/friend at risk of falling, 3) have you encountered any problems during your caring, 4) are there any risks in preventing your family member/friend from falling, and 5) have you received any advice, or support regarding fall prevention and from whom? Socio-demographic details such as age, relationship to the care recipient, time spent caring and the care recipients’ number of falls in the last 12 months were also collected.

As agreed by the carers, the interviews were audio-recorded. The researcher avoided interrupting the carers to allow them to speak freely about their concerns regarding their care recipient’s fall risk. However, the researcher would lead the carers back to the subject if they deviated from the topic, for example sharing only about the general caregiving burden. Sometimes, the questions were repeated, and probes were used to gain more insight or to find out if the carers understood what was being asked. The interviews lasted between 10 and 30 min.

### Data analysis

The audio-recorded interviews were transcribed verbatim and analysed by the first author MA using inductive content analysis [23]. Before the analysis, the transcripts were reviewed and verified against the audio recordings. The researcher first immersed himself in the data by reading the transcripts several times [24]. Codes describing content related to the study aims and interview questions were written in the transcripts during open coding [25]. These codes were transferred to the coding sheets and grouped to form categories. Similar categories were further combined to form higher order categories/ themes [24]. The first author met with two senior researchers (OB and AW) in the team every 2 weeks to review and refine the codes and themes formulated from the transcripts. The codes and themes were revised continuously as the study progressed to ensure that they fit the study data and addressed the study aims. After the interviews were completed, all transcripts were recoded using the final coding scheme. Summaries of the themes and sub-themes are illustrated in Tables 1 and 2.

**Table 1:** Summary of themes and sub-themes (Causes of fall concern)

Example of related sentences/phrases	Codes	Sub-themes	Themes
‘I guess I am always worried. She is always careful. I guess I am a bit worried that she may trip over a shoe or a floor mat’ (C17)	Concern about care recipient’s risk of fall	Possibility of fall	Carers’ perception of fall and fall risk
‘If he does fall, like you say like causing more damage to himself…breaking bones…making them pain’ (C15)	Concern about the consequence of fall	Consequence of fall	
‘Just a few bruises and scraped knees. There was nothing major, so we were fine’ (C14)	Little concern about minor injuries	Minor injuries	
‘I don’t worry much now because in the house he is using the walker…I feel that he’s got more stability’ (C4)	Confidence in care recipients’ balance	Confidence in balance	
‘If she wants to use it (the walker), she uses it. But I have told her if she had another fall, she will be going into care’ (C7)	Non-compliant to walking aid	Not listening to carers’ advice	Care recipients’ behaviour and attitude towards fall risk
‘The biggest challenge is getting through to both, they shouldn’t be lifting heavy weights’ (C9)	Continued with risky activities	Taking risks	
‘So, if I say things too often to her… she gets cranky and says stop pushing her in doing things’ (C16)	Upset with repeated reminders	Upset with repeated reminders	
‘My biggest concern is her not realising that she is getting older…She can’t do things like she used to be able to do’ (C7)	Unaware of fall risk	Unaware of fall risk	
‘My husband has starting to get a bit slower in his actions and his memory…due to his Parkinson’s disease’ (C13)	Cognitive and functional decline	Cognitive and functional decline	Care recipients’ health and function
‘Because she has arthritis in her knees and when she turns quickly her knees didn’t sort of go with her and she fell’ (C20)	Impaired gait and poor balance	Impaired gait and poor balance	
‘She is very good using her walker. Only that if she just gets up to answer the phone…a little quick, she might fall’ (C21)	Risk of falling when rushing to do things	Rushing to do things	
‘But our house is very flat now. Used to have a 2-storey house but luckily, we sold it last year. So, it’s all flat’ (C19)	Risk of falling when using the stairs	Presence of stairs	Care recipients’ living environment
‘He likes to just go out the street to get out of the house, I can’t let him go on his own’ (C10)	Risk of falling when going out alone	Going out alone	
‘I am only away for half an hour an hour, no problem. But any longer…we get a friend come in look after her’ (C19)	Risk of falling when alone at home	Alone at home	
‘Knowing that there is a close neighbour, it is very helpful because I think there’s always another person around’ (C9)	Feel reassured with support from neighbours	Support from neighbours

**Table 2:** Summary of themes and sub-themes (Management of care recipients’ fall risk)

Example of related sentences/phrases	Codes	Sub-themes	Themes
‘Maybe I don’t know everything that could be done, to prevent. That’s why I like to be around a lot more’ (C18)	Being around care recipient	Increase supervision	Fall prevention strategies used
‘I do telephone. Checking on her most of the days. I visit maybe three times a week’ (C17)	Telephone to check on care recipient	Calling the care recipient	
‘We really don’t go out much and leave each other. We do go shopping together, we prepare the meals together’ (C11)	Doing things together to reduce fall risk	Doing things together	
‘We have got railings outside the house for the steps to prevent falling. In the house we had modifications to the shower’ (C4)	Installing handrails to prevent falls	Home modification	
‘It happened so quickly. Out of the sudden she fell. It happened so quickly that you didn’t have time to support her’ (C2)	Fall is unexpected	Did not encounter such risk	Risk of preventing falls
‘I don’t try to assist because I could be injured as well. You should just let them fall, if they are going to fall’ (C4)	Afraid of getting injured when helping	Aware of physical limitation	
‘My daughter and some of the grandchildren, they help out at times. Especially with the yard and the bigger jobs’ (C8)	Help in manual activities by family members	Support from family	Support from family and friends
‘We have friends who put up rails at front and back for us. With the steps, and they made the steps smaller’ (C15)	Home modifications by friends	Support from friends	
‘It’s very tiring for me…we have the kids always promising to come in and help…and they got their own life’ (C10)	Lack of support	Lack of support	
‘When he had his first operation, the nurse in the emergency… gave me a piece of paper with what to do if he falls over’ (C5)	Given brochure on fall prevention	Information on fall prevention	Support from healthcare professionals
‘As part of the rehabilitation, there was physiotherapist instruction on what to do… to make sure you don’t fall’ (C6)	Physiotherapist advice in fall prevention	Support from allied healthcare professionals	
‘We were advised by the hospital about how to break the fall…Anyway, these were only after the fall’ (C2)	Advice on fall prevention after fall	Received support only after the fall	

Trustworthiness of the study findings was ensured following four criteria suggested by Lincoln and Guba which include credibility, transferability, dependability, and confirmability [26]. Confidence in the truth of study findings known as credibility was ensured by having two researchers (OB and AW) independently reviewing the congruence between selected content and themes generated. Any disagreement in the themes was discussed among the three researchers to reach consensus. Transferability, which refers to the applicability of study findings to other settings was determined by providing a detailed description of carers’ account for each theme and recruiting participants of different socio-demographic and caring relationships [25]. Dependability, which refers to stability of the findings was ensured by keeping an audit trail of the audio recordings, interview transcripts, coding sheets, and socio-demographic questionnaires to keep track of coding decisions and changes made to the codes during analysis [25]. To maintain objectivity of the findings known as confirmability, only carers willing to share about their concerns were recruited and the data were critically analysed by all three researchers.

## Results

The socio-demographic details of the carers are presented in Table 3. Of the 22 carers, 16 were females and 6 were males. Twelve were caring for their spouses, nine were caring for their parents, and one cared for a friend. The mean age of carers was 68 years (55 to 88) and care recipients was 81 years (61 to 99). The average length of caring was 7 years. Only eight carers had care recipients who had not fallen during the past year. Six carers reported that their care recipients sustained minor injuries, while six had sustained severe injuries, such as fractures, from the falls. Two care recipients had not sustained any injury. The carers were numbered C1–22 in this study. From the data analysis, four themes highlighted the causes of carers’ fall concern which include 1) carers’ perception of fall and fall risk, 2) care recipients’ behaviour and attitude towards fall risk, 3) care recipients’ health and function, and 4) care recipients’ living environment. Another four themes described the management of care recipients’ fall risk which include 5) fall prevention strategies used, 6) risk of preventing falls, 7) support from family and friends, and 8) support from healthcare professionals.

**Table 3:** Socio-demographic details of Carers

Interview method	Carer information								Care recipient (CR) information			
	Carer	Age range	Gender	Marital status	Employment status	Relationship to CR	Living with CR	Time spent caring(years)	Age range	Gender	Fall in past year	Fall injury
Telephone	1	70–79	Female	married	not working	spouse	yes	≥10	70–79	male	≥3	minor injury
Telephone	2	60–69	Male	married	not working	spouse	yes	0–4	60–69	female	1	severe injury
Face-to-face	3	70–79	Male	married	not working	spouse	yes	5–9	70–79	female	≥3	minor injury
Face-to-face	4	70–79	Female	married	not working	spouse	yes	5–9	80–89	male	1	severe injury
Face-to-face	5	80–89	Female	married	not working	spouse	yes	0–4	80–89	male	0	no injury
Face-to-face	6	70–79	Male	married	casual	spouse	yes	0–4	60–69	female	1	severe injury
Telephone	7	50–59	Female	married	not working	children	no	≥10	70–79	female	1	severe injury
Face-to-face	8	80–89	Female	married	not working	spouse	yes	0–4	80–89	male	2	minor injury
Face-to-face	9	60–69	Female	married	not working	children	no	0–4	80–89	female	1	no injury
Telephone	10	60–69	Female	married	not working	spouse	yes	0–4	70–79	male	2	minor injury
Telephone	11	80–89	Female	married	not working	spouse	yes	≥10	80–89	male	0	no injury
Telephone	12	70–79	Male	divorced	not working	friend	no	0–4	60–69	female	0	no injury
Telephone	13	70–79	Female	married	not working	spouse	yes	5–9	80–89	male	0	no injury
Telephone	14	60–69	Female	divorced	not working	children	no	0–4	90–99	female	≥3	minor injury
Telephone	15	60–69	Female	married	not working	spouse	yes	≥10	60–69	male	0	no injury
Telephone	16	50–59	Female	divorced	casual	children	no	5–9	80–89	female	0	no injury
Face-to-face	17	50–59	Female	married	part-time	children	no	5–9	80–89	female	0	no injury
Telephone	18	50–59	Female	Never married	casual	children	no	0–4	80–89	female	2	no injury
Face-to-face	19	70–79	Male	married	not working	spouse	yes	0–4	70–79	female	1	severe injury
Telephone	20	60–69	Female	married	part-time	children	no	≥10	90–99	female	1	severe injury
Telephone	21	60–69	Male	Never married	part-time	children	no	5–9	90–99	female	0	no injury
Telephone	22	60–69	Female	Never married	full-time	children	yes	5–9	90–99	male	≥3	minor injury

### Causes of fall concern

#### Theme 1: Carers’ perception of fall and fall risk

The perception of fall and fall risk varied among carers regardless of whether their care recipients had fallen previously. Many carers were constantly worried about the possibility of their care recipients falling again. For example, one older male carer whose wife had fallen more than three times over the past year commented that ‘You spend a lot of time worrying about where she is, if she is going to fall down the stairs, or fall in the shower…’ (C3). Another female carer also expressed concerns for her mother’s fall risk even though she had not fallen: ‘I guess I am always worried. She is always careful. I guess I am a bit worried that she may trip over a shoe or a floor mat’ (C17).

A few carers were concerned about the consequences of the fall causing additional harm to their care recipients and bringing an end to their independent living. This concern was illustrated by one female carer looking after her husband: ‘…if he does fall, like you say like causing more damage to himself, with arthritis and everything like that, breaking bones, or making them worst, and making them pain’ (C15).

In contrast, few carers were unconcerned about their care recipients sustaining minor injuries such as bruises and abrasions from the fall. When asked about the care recipient’s fall injuries, one female carer looking after her mother, who had sustained more than three falls in the past 1 year, replied that: ‘…just a few bruises and scraped knees. There was nothing major, so we were fine’ (C14). Many carers believed that their care recipients had a low risk of falling because they had employed paid home carers to assist them in their daily activities or with the household chores. The majority of carers highlighted that the presence of assistive devices such as walkers, shower chairs, and grab bars gave them confidence in helping with their care recipients’ balance. For instance, one older female spouse carer said that: ‘I don’t worry much now because in the house he is using the walker and he walks around he uses that all the time now. And when we go out shopping, he has another walker that he uses. So, I feel that he’s got more stability’ (C4). Another female spouse carer (81 years old) said that: ‘When he (her husband) is walking with his walker, he is very good. But if he holds onto the furniture and tries to walk, he could fall over then’ (C5).

#### Theme 2: care recipients’ behaviour and attitude towards fall risk

Theme 2 describes carers’ perception of their care recipients’ fall risk including actions which may increase carers’ fall concern. Carers, especially those looking after their parents said they had difficulty communicating fall risk to their care recipients. Several carers were concerned about the care recipients not listening to their fall prevention advice. This often resulted in feelings of frustration, stress, and helplessness. For example, one female carer, whose mother had sustained a fracture from her recent fall, commented that: ‘She should look after herself. If she wants to use it (the walker), she uses it. But I have told her if she had another fall, she will be going into care (institutional care) or I won’t be caring for her shoulder. She will hopefully use the walker’ (C7). Another female carer said that her parents refused to seek help when needed and continued with activities against advice, which put them at increased risk of falling: ‘The biggest challenge is getting through to both, they shouldn’t be lifting heavy weights. Mum chops wood sometimes and getting up on ladders’ (C9). Only one female spouse carer (C10) discussed about the difficulty of communicating fall risk to her husband. Instead, the majority of carers of spouses had concerns related to their care recipients’ health and functional status, and environmental risk factors.

In some cases when the care recipients were repeatedly reminded about their fall risk, they became upset and felt that their carers were trying to control them. One female carer looking after her mother (85 years old) said that: ‘So if I say things too often to her, oh what about doing this? What about doing that? Then she gets cranky and says stop pushing her in doing things’ (C16). There were several reasons suggested by carers about why the care recipients would not adhere to their fall prevention advice. Some carers believed that the care recipients were aware of their fall risk but resisted acknowledging their physical limitations by being dependent. This was explained by the previous carer that: ‘I think it’s the dignity thing that they still want their independence but it’s disappearing on them because of age and some of them can’t accept it where others their age accept it’ (C16). Another female carer felt her mother (79 years old) was unaware of her fall risk and therefore did not take any measures to protect herself: ‘…my biggest concern is her not realising that she is getting older. I must keep reminding her she is getting older. She can’t do things like she used to be able to do then’ (C7).

#### Theme 3: care recipients’ health and function

The care recipients’ cognitive and functional decline leading to an increased risk of falling was highlighted when carers discussed fall concern. This was often associated with issues of ageing, or other pre-medical conditions, such as dementia and Parkinson’s disease. For example, one older female carer (C13) noticed that her husband (84 years old) had started to ‘get a bit slower’ in his actions and memory due to his Parkinson’s disease. A younger female carer (C16), was extremely concerned about her mother (85 years old) falling, as she realised that her mother was starting to lose her memory and would forget what she was saying, or supposed to be doing. One female carer, looking after her mother (99 years old), described the issue of cognitive decline as unavoidable and likely to worsen over time: ‘I think that will pretty go on for as long as she lives because she just forgets, forgets more with some things’ (C14).

Impaired gait and poor balance were emphasised by several carers when talking about their care recipients’ fall risk. In some instances, it was also the main reason for the fall. One female carer, who was looking after her mother (92 years old), described an event that: ‘She turned quickly and her knees kind of didn’t because she has arthritis in her knees and when she turns quickly her knees didn’t sort of go with her and she fell broke her hip’ (C20).

Furthermore, a few daily activities such as showering, getting up from bed, or a chair, and using the stairs, were highlighted by carers as potential risks in causing a fall. This prompted carers to try to supervise and assist their care recipients in these activities. There were also concerns that the care recipients rushed to do things, or forgot to use their walking aid, especially when carers were not around to remind them, resulting in a fall. One male participant caring for his mother commented that: ‘She is very good using her walker, which is, you know is great. Only that if she just gets up to answer the phone, or something and is a little quick, that’s all, that she might fall in her unit’ (C21).

Besides increasing carers’ fall concern, the issue of the care recipients’ health and function may affect the overall caregiving process and level of support required. This was illustrated in one female carer’s account: ‘Since mum has dementia, I have become more involved with her care. I don’t just think of her risk of falling, but I actually think of everything else and that she’s safe’ (C18).

#### Theme 4: care recipients’ living environment

Several carers discussed concern regarding their care recipients using stairs and falling at home. This concern was often associated with the care recipients’ health issues such as syncope or functional decline. One female carer mentioned that: ‘if she (her mother) is going to have another one of these episodes where she blacks out a little bit, if she is going to be going down the stairs, that’s a big risk and there is no hand rails’ (C16). A few carers also expressed relief that there were no stairs at home, especially after their care recipients had sustained an injury from a fall. One older male carer whose wife had broken her hip from a fall said that: ‘But our house is very flat now. Used to have a 2-storey house but luckily, we sold it last year. So, it’s all flat’ (C19). Other concerns expressed by carers included tripping over uneven floors, or objects, walking on a slope, or a wet surface. This was highlighted in one female carer’s account: ‘my bathroom is lower than the floor. About a good 6 inches lower than the floor. So, you’re not only stepping into a bath, you step into a drop (C10)’.

The concerns about care recipients falling outside the home were similar to those in the home. Nevertheless, these outdoor concerns were often considered inevitable and difficult to control. For example, one female carer whose mother (92 years old) was living alone said that: ‘we have taken all the precaution we need to, like removing mats and making the house as safe as possible. But there’s probably not a lot we can do especially when she is going out of the house and unless she avoids using the steps at all’ (C20). A few carers were even reluctant to let their care recipients to go out alone. For instance, one female carer whose husband had fallen twice last year added that: ‘I won’t put him alone no, and I won’t let him go anywhere on his own, no. Like if he wants to go. He likes to just go out the street to get out of the house, I can’t let him go on his own’ (C10).

Many carers expressed concern about their care recipients falling when alone at home. A few carers would only leave their care recipients alone for a short period of time or get someone else to look after them. Others could not leave their care recipients alone at all. For carers who were not living with their care recipients, many were reassured that they are living near to them, or their care recipients have had neighbours to look out for them. This was highlighted by one female carer who was looking after her mother: ‘Knowing that there is a close neighbour, it is very helpful because I think there’s always another person around’ (C9).

### Management of care recipients’ fall risk

#### Theme 5: fall prevention strategies used

Carers identified various strategies to prevent their care recipients from falling which included increasing supervision, assisting with daily activities, providing support during mobility, or encouraging physical activity. Close monitoring of the care recipients was the most commonly used method in situations such as making sure that they use their walking aid and being around when they shower. For example, one female carer chose to spend more time with her mother who was suffering from dementia because of the lack of fall prevention knowledge: ‘Maybe I don’t know everything that could be done, to prevent. That’s why I like to be around a lot more’ (C18).

Most carers regularly called to check on their care recipients if they were not living with them. For example, one female carer said that: ‘I do telephone. Checking on her (her mother) most of the days. I visit maybe three times a week and that will generally include an outing, we go out like the social occasion, coffee or lunch’ (C17). Another female carer looking after her father with mobility impairment also said that: ‘I ring him several times a day to make sure he’s okay’ (C22). Additionally, one female carer (C16) had encouraged her mother with an impaired gait to carry a phone with her at home, just in case she fell and needed to call someone. A majority of carers also mentioned helping out with the difficult chores such as preparing meals, doing grocery shopping, cleaning the house, and doing laundry in an effort to reduce the fall risk of their care recipients.

Several carers, who were looking after their spouses, were at risk of falling themselves. This group described strategies such as looking out for each other’s risk, planning and doing things together. For instance, one older female spouse carer said that: ‘…we really don’t go out much and leave each other. We do go shopping together, we prepare the meals together, and we do the dishes together. So, we were 85% of the time we were in our own home’ (C11). However, regardless of the caring relationship, the majority of carers discussed about making changes to the environment to prevent their care recipients from falling. This included installing handrails, replacing the bathtub with a shower, levelling the floor, and removing of carpets or mats. This was highlighted in one female spouse carer’s account: ‘…we have got railings outside the house for the steps to prevent falling. In the house we had modifications to the shower and railings in the shower’ (C4).

#### Theme 6: risk of preventing falls

When asked about the risks of preventing their care recipients from falling, only one carer (C1) mentioned sustaining an injury (i.e. sprain) while trying to assist her husband get up from the fall. Most carers claimed they had not encountered this problem. For example, one younger male spouse carer mentioned that: ‘It happened so quickly. Out of the sudden she fell. It happened so quickly that you didn’t have time to support her or anything like that’ (C2). A few carers were aware of their physical limitation and the risk of sustaining an injury when assisting their care recipients during the fall. For instance, one older female spouse carer said that: ‘I don’t think he would be able to get up on his own. I don’t try to assist because I could be injured as well. You should just let them fall, if they are going to fall’ (C4). Another female carer who was looking after her mother when the fall occurred said that: ‘I can’t hold her if she falls. I think there was one fall which I was holding onto her arm. I had to let go because when she fell, it was dead weight and I would have landed on top of her’ (C14).

#### Theme 7: support from family and friends

Several carers received support from their family and friends in fall prevention, which included assistance in activities requiring manual handling, monitoring of care recipients, and home modification. As previously mentioned, some older spouse carers were also at risk of falling due to functional decline and increasing age. Support in undertaking complex activities may help these older carers better manage their care, minimise the risk of falling for both carers and their care recipients, and alleviate their fall concern. For example, one female spouse carer said that: ‘My daughter and some of the grandchildren, they help out at times. Especially with the yard and the bigger jobs’ (C8). Another female carer looking after her mother mentioned that: ‘Since the fall, I visit my mother every day, my sister does some nights, my niece does some nights, and another niece does some nights’ (C14). One female spouse carer (C15) said that her friends came to install hand rails and modify the stairs (made smaller steps) in her house. This support helped mitigate her disappointment with the delay in professional help from the hospital and community disability services.

In contrast, some carers expressed frustration with the lack of support from their family members in the general provision of care and activities which may be beyond the physical limitation of carers and were potential falling risks. For example, one female spouse carer (67 years old) said that: ‘It’s very tiring for me. So, it’s a bit of everything. He doesn’t listen, we have the kids always promising to come in and help, mow the lawn, and things like that, and they got their own life’ (C10). Similarly, another younger female carer looking after her mother also said that: ‘My sister and her children sometimes visit. But they just go and have a chit chat and leave, while I used to spend the whole day there and do stuff’ (C16).

#### Theme 8: support from healthcare professionals

The majority of carers did not receive any fall prevention information from healthcare professionals. Among those who had received advice on the management of their care recipients’ fall risks, information was delivered in the form of brochures provided by nurses in the hospital. For example, one older female spouse carer said: ‘When he had his first operation, the nurse in the emergency took me into a quiet room and she gave me a piece of paper with what to do if he falls over and what to do if he’s disoriented. That was very helpful’ (C5). A few carers also reported that their care recipients had received services from occupational therapists for home assessment and modification, and physiotherapists for body strengthening exercises to prevent falls. For instance, one male spouse carer said that: ‘As part of the rehabilitation, there was physiotherapist instruction on what to do and how to look after yourself and what muscles to build up to make sure you don’t fall’ (C6).

While the majority of carers who had received advice and support on fall prevention from healthcare professionals were satisfied with these services, one male carer whose wife had sustained multiple fractures from a fall felt that this advice had come too late: ‘We were advised by the hospital about how to break the fall if things like that were to happen again, like if it is possible. Anyway, these were only after the fall’ (C2). He added that: ‘My wife, prior the hospitalisation only had two falls. She had talked to the doctor about it. The doctor said well that happens when you get old. That wasn’t very good advice’. Another female carer (C17) wanted to know more about fall prevention but did not know who to seek advice from since her mother has not fallen.

## Discussion

This study explores the complex relationship between carers and their concerns for their older care recipients who were at risk of falling at home. The findings contribute to the literature in two major ways. First, the study reveals four main themes contributing to carers’ fall concern, which include carers’ perception of fall and fall risk, care recipients’ behaviour and attitude towards fall risk, their health and function, and living environment. Second, the findings highlight the carers’ level of knowledge and strategies used in preventing their care recipients from falling. Since carers such as family members and friends are often the main support person for older people at home, these findings could create the potential for collaboration between healthcare providers and carers to develop a comprehensive fall prevention programme tailoring to both the care recipients and their carers.

A systematic review and meta-analysis of exercise interventions found that exercises including Tai Chi, yoga, balance training, strength and resistance training have only small to moderate short-term effect in reducing fear of falling among older people, but no significant long-term effect [27]. Therefore, the inclusion of carers in fall prevention programmes; improving their understanding of fear of falling and providing strategies to appropriately support their care recipients, could potentially reduce care recipients’ fear of falling and maintain this effect over time. This hypothesis is supported by a study where care recipients reported greater satisfaction in managing the fear of falling, especially when strategies to minimise their fall risk and fear of falling were supported by family members [28]. These strategies included providing instrumental support such as installing handrails and obtaining assistive devices to increase their independence, or emotional support such as discussing fall concern with the care recipients. Another study found that care recipients with lower social support demonstrated significant increase in falls self-efficacy after a fall prevention programme (including group exercises) and at 5-months follow-up [29].

The findings from this study provide an understanding of how carers see potential contributory factors to the risk of their care recipients falling at home. Previous research shows carers of fallers are concerned about the likelihood of their care recipients falling again [8, 9, 12, 14]. This study shows that carers of non-fallers are equally concerned about the risk of their care recipients falling. Moreover, carers’ concern for their care recipients’ risk of falling is not only due to ageing and intrinsic factors such as cognitive and functional decline, impaired gait, and poor balance [14]. Factors, such as the presence of stairs, the care recipients living alone, or support from neighbours can either increase or mitigate their concern.

A small group of carers in this study were not concerned about their care recipients sustaining minor injuries as a result of the falls. This finding corresponds with another study, which found that carers have little concern about their care recipients with Parkinson’s disease falling, as it was accepted as part of the disease progression [30]. However, care recipients were at risk of entering long-term care if they sustained severe injuries from the falls. Therefore, it is important for healthcare professionals to assist carers in developing accurate appraisals of their care recipients’ fall risk [31].

Another finding is the possible influence of the caring relationship on carers’ fall concern and strategies used to prevent their care recipients from falling. As with other studies, carers reported difficulty communicating fall risk to their care recipients as they refused to follow carers’ advice [14], walked without their walking aid [13, 32], and took risks by not seeking help [12]. This concern was mainly discussed by carers looking after their parents. It seems that carers in the child-parent relationship may have a greater disparity in appraisals of fall risk compared to those in spousal relationships. Carers who were looking after their spouses were about the same age and may have similar fall risk as their care recipients. Therefore, they could be unaware of their fall risk and were less likely to take appropriate measures to prevent their care recipients from falling.

In addition to differences in perceptions of fall concern among carers, the choice of fall prevention strategy also varied among carers who were looking after their parents and those looking after their spouses. Our study revealed that supervision in the form of close monitoring, regular visiting and contacting their care recipients by telephone are commonly used by female carers looking after their parents to prevent a fall. This finding is different from another study, which found that sons but not the daughters took on a more ‘protective’ role of supervision in preventing their mothers from falling [22]. The latter study also found that daughters were involved in working together with their parents to provide falls supervision. In contrast, the fall prevention strategies used by spouse carers in our study tended to be more collaborative, such as looking out for each other’s fall risk and doing things together. The difference in fall prevention strategies used may be attributed to the power imbalance between carers and their care recipients, with younger carers having more control in the caring relationship. However, future research is needed to ascertain the difference in management of fall risk between spouse carers and those caring for their parents.

This study found that support from family and friends is important for carers to cope with the management of their care recipients’ fall risk. However, not all carers had external support and some also did not have adequate knowledge about preventing falls. As found in other studies, the use of inappropriate strategies such as over-protection and increased supervision could lead to stress, exhaustion, and social isolation among carers [12–14]. These strategies may also increase the risk of care recipients falling. For example, calling the care recipients several times per day could be a fall risk if care recipients with mobility impairment try to answer the telephone. Therefore, it is important that carers are supported when implementing fall prevention strategies for their care recipients such as improving their awareness of fall risk and providing them with strategies to prevent falls.

### Implications for practice

The increasing onus on carers for the implementation of sustainable fall prevention interventions at home [33] means it is important that healthcare professionals develop fall prevention programmes which are inclusive of carers. To date, fall prevention strategies are designed only for care recipients (older people) targeting falls [34] and their fear of falling [27]. An individualised approach for carers when implementing fall prevention strategies is recommended. This would mean identifying the impact of care recipients’ fall risk on their carers and their management strategies in preventing care recipients from falling. Healthcare professionals could assist carers to recognise their care recipients’ fall risk, provide counselling for fall concern, and discourage potentially harmful strategies such as increasing supervision or restricting outings. Positive strategies already in use, such as doing household chores together (with care recipients) and seeking help from family and friends should be reinforced. Future fall prevention programmes should also cater to carers whose care recipients have not fallen so that they are aware of their care recipients’ risk of falling.

Regarding implications for research and practice, this study has generated a pool of measurable items which will be used to develop an instrument for assessing carers’ fall concern. This instrument will allow healthcare professionals to better understand the impact of fall risk on carers and identify the association between fall concern and other psychological factors. For example, if anxiety and depression are associated with increased carers’ fall concern, healthcare professionals need to be mindful of this during the assessment of care recipients’ fall risk before developing an individualised fall prevention programme for carers and their care recipients. The multiple factors associated with carers’ fall concern also highlights the need for a multidisciplinary team to manage the physical, psychological and social needs of carers and their care recipients when preventing falls at home.

### Limitations

There are several limitations of this study. One of the limitations was the length of the interviews lasted only between 10 and 30 min. However, we stopped recruiting when no new themes emerged from the qualitative data. The study was conducted with a small sample of carers in one state of Australia and is not representative of the larger population of carers, such as those living in nearby Asia, or the rest of Australia. Nevertheless, it is likely that the findings would be common to other carers looking after older people at home. The use of telephone interviews prevents direct observation of carers’ gestures and actions which could be used for comparison with the interview data during analysis to determine the accuracy of information shared [35]. Furthermore, the lack of prolonged engagement and personal contact in telephone interviews may also prevent the researcher from building trust and rapport with carers, which is important for obtaining meaningful information. However, we found that this option gave carers more flexibility to participate in the interview at a time of their convenience and allowing them to feel more relaxed and to share their private concerns freely [35].

The researcher who conducted the interviews was also involved in the data analysis. To ensure trustworthiness of the findings, investigator triangulation was achieved by the involvement of two other researchers (OB and AW) to validate the categories derived [26]. This study was motivated by the first author’s experience of working with older people who were admitted to the hospital for recurrent falls and interaction with their anxious carers. His prior experience interviewing carers to develop discharge plans for their care recipients (older people) may strengthen his ability to probe in-depth about carers’ concern. However, this experience may also affect the researcher’s objective interpretation of the findings [36].

## Conclusion

This study reveals that concern about care recipients at risk of falling may be attributed to four themes (i.e. carers’ perception of fall and fall risk) involving carers and their care recipients. Different strategies were used for managing the care recipients’ fall risk, and some carers had extra support from family and friends, or healthcare professionals in falls prevention. An individualised fall prevention programme catering to carers is encouraged in assisting carers to accurately identify their care recipients’ fall risk, cope with fall concern, and implement strategies for preventing falls. This could potentially improve carers’ confidence in managing their care recipients’ fall risk and, potentially reduce the incidence of falling among older people, and allowing them to stay at home longer.

## Data Availability

The data generated during the current study are not available due to ethics requirement that the interview transcripts are to be kept in password protected files and accessible only by the researchers.

## Abbreviations

Carers NSW: Carers New South Wales
HMRI: Hunter Medical Research Institute
